# Editorial: Expert opinions and viewpoints in nuclear medicine

**DOI:** 10.3389/fmed.2026.1899911

**Published:** 2026-07-07

**Authors:** Giorgio Treglia

**Affiliations:** 1 Division of Nuclear Medicine, Ente Ospedaliero Cantonale, Bellinzona, Switzerland; 2 Faculty of Biomedical Sciences, Università della Svizzera Italiana, Lugano, Switzerland; 3 Faculty of Biology and Medicine, University of Lausanne, Lausanne, Switzerland

**Keywords:** diagnosis, imaging, nuclear medicine, oncology, PET, prognosis

The Research Topic “*Expert Opinions and Viewpoints in Nuclear Medicine*” includes four articles (one study protocol and three reviews) that are mainly focused on Positron Emission Tomography/Computed Tomography (PET/CT) in oncology.

Positron emission tomography (PET) is currently one of the most widely employed imaging modalities in oncologic nuclear medicine. Among the available radiopharmaceuticals, Fluorine-18 fluorodeoxyglucose ([18F]FDG), which reflects glucose metabolism, remains the most frequently used tracer in clinical practice. [^18^F]FDG PET is routinely used for tumor staging, restaging, and treatment response evaluation in a variety of malignancies characterized by increased glucose metabolism. Nevertheless, its role in certain cancer types is still under investigation.

In this context, the study protocol presented by Urso et al. focused on the application of [^18^F]FDG PET/CT in muscle-invasive bladder cancer (MIBC). Conventional MIBC staging relies primarily on standard imaging techniques, which often show limited sensitivity for the detection of nodal and distant metastatic disease. Recent evidence has suggested that [^18^F]FDG PET/CT may offer additional value in both staging and treatment monitoring of MIBC. This prospective MIBC-PET study enrolled patients with newly diagnosed, histologically confirmed, high-grade MIBC and compared [^18^F]FDG PET/CT with contrast-enhanced CT (ceCT). Participants underwent baseline imaging with both modalities, followed by repeat examinations after two cycles of neoadjuvant therapy. To reduce the impact of urinary tracer activity, the PET protocol incorporated both standard delayed imaging and an early dynamic pelvic acquisition. The study aimed to assess the diagnostic accuracy of [^18^F]FDG PET/CT compared with ceCT for disease staging and to evaluate its ability to predict pathological response to neoadjuvant chemotherapy. Additional objectives included validating the role of early dynamic PET imaging for local staging and exploring artificial intelligence-driven predictive models (Urso et al.).

The review by Albano et al. examined the clinical significance of semiquantitative [^18^F]FDG PET/CT parameters in lymphoma. These imaging biomarkers capture various aspects of disease biology, including tumor burden, disease dissemination, body composition, and textural heterogeneity. Although many of these metrics have demonstrated promising prognostic and predictive capabilities, their broader clinical implementation is hampered by a lack of standardized acquisition, analysis, and reporting methodologies. The review summarized the current evidence supporting the value of these parameters while highlighting the challenges that must be addressed before their routine adoption in clinical practice (Albano et al.).

Another emerging PET-derived biomarker is maximum tumor dissemination (Dmax), a semiquantitative parameter defined as the greatest distance between the two most distant hypermetabolic lesions identified on PET/CT. Tasevski et al. conducted a systematic review to evaluate the role of Dmax across different oncologic settings. Thirty-seven studies were included, with lymphoma representing the most extensively investigated malignancy, followed by prostate, lung, and breast cancers. Despite substantial methodological heterogeneity, the majority of studies reported a significant association between Dmax and clinical outcomes, including overall survival (OS) and progression-free survival (PFS). Furthermore, combining Dmax with other PET-derived metrics, particularly metabolic tumor volume (MTV), appeared to improve patient risk stratification. However, the authors emphasized that several methodological and standardization issues remain unresolved and must be addressed before widespread clinical implementation of Dmax (Tasevski et al.).

Finally, Strauff et al. provided a comprehensive overview of the opportunities and challenges associated with the clinical translation of radiopharmaceuticals for pediatric applications. The authors underlined that current regulatory frameworks have largely been developed for adult populations, resulting in a significant gap in pediatric radiopharmaceutical research. Emerging technologies, including novel imaging tracers, improvements in scanner sensitivity, and artificial intelligence-based approaches, may help overcome some of these limitations. Moreover, stronger international collaborations, multicenter initiatives, and data-sharing strategies are considered essential to accelerate the development and validation of radiopharmaceuticals in children. Greater harmonization of regulatory requirements would further support ethically sound pediatric research and facilitate evidence-based decision-making (Strauff et al.).

Overall, the contributions included in this Research Topic highlight several evolving applications of PET/CT, particularly in oncology, and underscore its growing relevance in the era of precision and personalized medicine.

